# Implications of Incomplete Immunostaining in Membranous Lupus Nephritis

**DOI:** 10.34067/KID.0000000000000191

**Published:** 2023-07-27

**Authors:** Kana N. Miyata, Cynthia C. Nast

**Affiliations:** 1 Division of Nephrology, Department of Internal Medicine, Saint Louis University, Saint Louis, Missouri; 2 Department of Pathology, Cedars-Sinai Medical Center, Los Angeles, California

**Keywords:** complement, glomerular disease, histopathology, immune complexes, lupus nephritis, membranous nephropathy

Membranous lupus nephritis (MLN), International Society of Nephrology/Renal Pathology Society lupus nephritis class V, accounts for 10%–20% of cases of lupus nephritis.^1^ Although current evidence suggests that pure MLN has a more favorable renal outcome compared with proliferative or mixed forms of lupus nephritis, MLN is not a benign disease, with the reported incidence of ESKD as high as 10%–37% in 10 years.^2^ Classically, the histological findings of pure MLN include full-house (FH) immunofluorescence with glomerular deposits of IgG, IgM, IgA, C1q, and C3 in the subepithelial and almost always mesangial areas. However, up to 48% of kidney biopsies from patients meeting clinical criteria for systemic lupus may have MLN lacking one or more of these immune reactants.^3^ It is unknown whether the absence of FH immunofluorescence has clinical implications in patients with MLN.

In this issue of *Kidney360*, Ye *et al.* address this knowledge gap by performing a retrospective multicenter study of kidney biopsies with a diagnosis of MLN.^4^ They compared the clinical and pathologic features and outcomes of 113 patients with initial MLN kidney biopsies showing FH (≥1+ IgG, IgM, IgA, C1q, and C3), non–full house (NFH; ≥1+ IgG, 0 or trace IgA, IgM and C1q, any C3), or intermediate (INT; not meeting FH or NFH criteria) immunofluorescence staining. NFH biopsies were a minor subset, accounting for 12.6% (16 of 127) of all MLN cases from one institution and 13.4% of initial MLN biopsies. Patients with NFH versus FH tended to be older at the time of SLE diagnosis, and the average time from lupus diagnosis to initial kidney biopsy was significantly longer for NFH (8 years) versus FH (3.16 years, *P* = 0.013) and INT (4.05 years, *P* = 0.033). Interestingly, patients with NFH had significantly higher serum creatinine levels at the time of biopsy (1.43 versus 0.71 mg/dl, *P* = 0.017) but fewer extrarenal systemic manifestations of lupus compared with FH MLN. No differences in onset during childhood, autoantibody prevalence, decreased complement levels, degree of proteinuria, treatment of lupus, or progression to ESKD or death were found, although power was limited by small patient numbers reaching the outcome measures. Analysis of the histologic features showed that all cases had low chronicity with no differences among the biopsies in the modified National Institutes of Health chronicity index, in the degree of acute tubular injury or in the Ehrenreich and Churg stage of membranous nephropathy. By contrast, the intensity of glomerular C3 staining was significantly lower in NFH (1.16+) versus FH (2.38+) and INT (1.94+) biopsies (*P* < 0.0001 and *P* = 0.001, respectively).

The NFH biopsies were from older patients and were associated with a higher serum creatinine level at biopsy, which had no clear explanation. These patients had a longer lupus course before biopsy with fewer systemic manifestations of lupus. This suggests a milder disease course associated with NFH MLN, although this would be supported further by providing follow-up serum creatinine levels or slopes of decline in the different patient groups. The reduced systemic disease activity and possibly more benign course may be related to attenuation of complement-mediated injury in NFH, evidenced by significantly reduced C3 staining in the glomerular deposits. A recent report from China,^5^ which analyzed a single-center cohort of 217 active lupus nephritis biopsies including 47 pure MLN cases (21.7%), found that glomerular IgM deposition positively correlated with C3 deposition and inversely correlated with plasma levels of C3 and complement factor H. Therefore, the decreased C3 staining in NFH MLN may be, in part, related to reduction in IgM deposition. A study examining the deposition of C4d in lupus nephritis biopsies showed that in a small number of cases, NFH MLN was associated with strong C4d and absent C3 and C1q staining, suggesting a role for activation of the lectin complement pathway.^6^ Kidney biopsy C3 staining without C1q or C4, indicating alternative complement pathway activation in patients with lupus nephritis, has been associated with disease progression and worse renal outcomes.^7^ It is possible that reduced C3 deposition in MLN may indicate less alternative pathway activation with delayed and/or milder kidney injury. It also is possible that there are other distinct underlying immunological mechanisms of disease associated with NFH immune complex deposition.

This study generates new perspectives and raises questions relating to MLN. It is unknown whether NFH MLN transitions to FH MLN or to proliferative lupus nephritis. Transition from MLN to proliferative lupus nephritis occurs in one-third of the cases, and this is especially important as a prognostic factor. It is of clinical interest to determine whether differences in immunostaining patterns contribute to or predict subsequent class transition. Exostosin (EXT) 1 and EXT2 proteins are recently discovered putative membranous nephropathy antigens observed in 34.6% of patients with MLN,^8^ and EXT 1/2 positivity is associated with a milder clinical course of MLN.^9^ Because NFH MLN also seems to have a more benign disease presentation and course, it would be of interest to know what percentage of these cases stain for EXT 1/2. Similarly, neural cell adhesion molecule 1^10^ and transforming growth factor *β* receptor 3^11^ are target antigens in a subset of MLN cases. Neural cell adhesion molecule 1–positive MLN has been associated with FH staining in 40% (8 of 20), NFH in 20% (4 of 20), and INT in 40% (8 of 20),^10^ while transforming growth factor *β* receptor 3–positive MLN cases have FH staining in 47% (8 of 17).^11^ Therefore, more than half of the MLN cases associated with these antigens have NFH or INT staining, and it remains to be seen whether this indicates specific mechanisms of injury or provides important clinical correlations.

In summary, Ye *et al.* have provided useful observations regarding NFH MLN, which may represent a distinct subcohort of patients. Larger studies are needed to evaluate complement activation, specific antigens associated with MLN, disease progression, and outcomes in these patients to determine underlying mechanisms of kidney injury and the potential diagnostic utility of NFH staining for optimizing treatment and predicting prognosis.

## Disclosures

C.C. Nast reports the following—consultancy: BioCryst Pharmaceuticals and advisory or leadership role: BioCryst Pharmaceuticals. The remaining author has nothing to disclose.
